# Robotic-assisted hip and knee replacement in NHS scotland: trends and efficiency implications (2020–2024)

**DOI:** 10.1007/s11701-025-03086-7

**Published:** 2026-01-09

**Authors:** Wissem Tafat, Marcin Budka, David McDonald, Robert G. Middleton, Findlay Welsh, Thomas W. Wainwright

**Affiliations:** 1 Orthopaedic Research Institute (ORI), Bournemouth University, Poole, UK; 2 Faculty of Science and Technology, Bournemouth University, Poole, UK; 3 Centre for Sustainable Delivery, Golden Jubilee National Hospital, Clydebank, UK; 4 University Hospitals Dorset NHS Foundation Trust, Bournemouth, UK; 5 Golden Jubilee National Hospital, Clydebank, UK

**Keywords:** Hip replacement, Knee replacement, Robotic surgery, Surgical efficiency, Surgical outcomes, Operative time

## Abstract

Robotic-assisted hip and knee replacement has been increasingly adopted in orthopaedic practice, offering potential advantages in surgical precision and workflow consistency. However, its efficiency compared with conventional manual methods in real-world NHS practice remains debated. This retrospective observational study analysed routinely collected data from the Arthroplasty Rehabilitation in Scotland Endeavour (ARISE) programme, including patients undergoing unilateral primary hip or knee replacement between 2020 and 2024. Robotic-assisted status, type of surgery, and operative duration were examined. Uptake of robotic-assisted hip and knee replacement was assessed annually, and comparative analyses evaluated operative times between robotic-assisted and manual procedures. Robotic-assisted hip and knee replacements increased from 101 procedures in 2020 to 1164 in 2024, representing a tenfold rise. Despite this growth, robotic cases accounted for fewer than 10% of all hip and knee replacements, with uptake concentrated in a small number of centres. Median operative durations were similar between robotic-assisted and manual procedures for both hips and knees. However, robotic surgeries demonstrated narrower interquartile ranges and fewer outliers. Knee replacements showed consistently higher adoption than hip replacement across the study period. Robotic-assisted hip and knee replacement has expanded rapidly in NHS Scotland, though access remains uneven across hospitals. Operative efficiency is comparable to conventional methods, but improved consistency may offer service-level benefits such as more predictable theatre scheduling. Wider adoption will require strategic investment in infrastructure, training, and equitable resource distribution to maximise the potential benefits of robotic-assisted technology within the NHS.

## Introduction

Total hip and total knee replacement surgeries are among the most common and successful orthopaedic procedures performed, offering substantial improvements in mobility and quality of life for patients with hip and knee osteoarthritis [1]. In the National Health Service (NHS) Scotland, as in other parts of the United Kingdom (UK), the demand for these procedures continues to grow due to an ageing population and the subsequent increased prevalence of osteoarthritis [2, 3].

In recent years, robotic-assisted surgery has emerged as a transformative assistive technology in healthcare. Between 2016 and 2023, there was a 524% increase in robot-assisted surgeries across the NHS and independent sectors in the UK, which demonstrates a substantial rise in adoption [4]. Robotic systems can help to enhance surgical precision, optimise implant positioning, and potentially improve patient outcomes. Unlike traditional manual surgery, some robotic systems assist the surgeon by providing 3D planning and real-time guidance during the operation [5]. It is important to note that robotic-assisted arthroplasty does not represent a single uniform technology, as systems differ substantially in their underlying design and intra-operative workflow. Some platforms are image-based, incorporating pre-operative imaging and haptic guidance, whereas others are non–image-based systems that assist the surgeon by positioning instrumentation such as cutting blocks.

While this technology is associated with benefits such as improved alignment, fewer complications, and faster recovery, it is also accompanied by concerns about cost-effectiveness and operational efficiency in real-world settings [6]. Within the orthopaedic sector, the incorporation of robotic-assisted technology has ushered in a new era characterised by greater precision and efficiency. From hip and knee joint replacements to spinal procedures, robotic systems have transformed the landscape of orthopaedic surgery, offering unparalleled accuracy and consistency.

Within the context of the NHS, where resources and operating time are critically managed, understanding the implications of adopting robotic-assisted surgery is particularly important because, despite its growing popularity, a common perception remains that these types of surgeries may be more time-consuming than conventional methods [7, 8]. Given the significant investment required to purchase robotic-assisted surgery systems and the pressure on surgical waiting lists, it is essential to evaluate the practical impact of this technology.

This study aims to investigate the adoption and efficiency of robotic-assisted total hip and knee replacements within NHS Scotland. By analysing routinely collected data, we assess how the adoption of robotics has evolved over time and whether robotic-assisted hip and knee replacement is associated with a longer operative time compared to manual procedures.

### Research questions

- What has been the uptake of robotic-assisted hip and knee replacement in NHS Scotland from 2020 to 2024?
- Does robotic-assisted hip and knee replacement take longer than standard (manual) operations?

## Methods

### Description of data sources

This retrospective observational study was conducted on routinely collected surgical data from NHS Scotland, specifically from the Arthroplasty Rehabilitation in Scotland Endeavour (ARISE) programme. ARISE is a national initiative aimed at enhancing adherence to standardised enhanced recovery pathways for hip and knee arthroplasty procedures across Scotland. The programme focuses on improving patient outcomes by promoting evidence-based perioperative care protocols and reducing variability in clinical practice [9].

The dataset included anonymised demographic, clinical, and perioperative information for patients who underwent hip or knee replacement procedures between January 1, 2020, and December 31, 2024, across multiple NHS hospitals. Only unilateral primary joint replacements were included in the analysis, and therefore, bilateral and revision replacements were excluded, as they take longer than primary unilateral procedures and could therefore bias comparisons in the analysis.

Access to the data was granted through the ARISE project. Ethical clearance for this study was granted by Bournemouth University on March 2, 2025, following prior approval from the Golden Jubilee National Hospital Audit Committee on January 29, 2024. A full NHS ethics review was not required because the project used anonymised secondary data supplied by NHS Scotland. All data were fully de-identified in line with data protection regulations, and no information that could identify individual patients or hospitals was included in the analysis. The study was carried out in accordance with recognised ethical principles and reported following the STROBE guidelines [10].

### Variable definitions

1. Manual vs. robotic-assisted hip and knee replacement: The variable surgical_approach was used to determine robotic status. A value of “Yes” indicated a robotic-assisted procedure, while “No” indicated a standard manual operation.
2. Type of Surgery (Hip vs. Knee): A new variable, type, was derived from the one-hot encoded operation fields and classified each case as either a hip or knee procedure.
3. Surgery Duration: The total duration of surgery was calculated as the time in minutes from the recorded start of surgery (knife-to-skin) to the time the patient entered the recovery area. For quality control, only cases with durations between 30 and 180 min were included in the final analysis to exclude outliers and data entry errors.

### Analytical approach

1. Uptake of robotic-assisted hip and knee replacement: The uptake of robotic-assisted hip and knee replacement was analysed by calculating the annual counts and percentages of robotic-assisted versus manual procedures from 2020 to 2024. These proportions were also categorised by operation type.
2. Surgical Duration Analysis: Comparative analysis was conducted to assess differences in surgical duration between robotic-assisted and manual procedures. Descriptive statistics, including mean, median, and standard deviation, were calculated. Boxplots were used to visualise the distribution of surgical duration.

## Results

Table 1 shows the distribution of surgical procedures across the study years. Primary hip and knee arthroplasties consistently made up the majority of operations, together accounting for over 90% of cases annually. Bilateral procedures (THA, TKA, or unicompartmental knee) were rare, each representing < 1% of annual totals.

**Table 1:** Percentage distribution of surgical operation types by year (2020–2024)

Operation surgery year	Bilateral THA	Bilateral TKA	Bilateral Unicompartmental Knee	Primary THA	Primary TKA	Revision THA	Revision TKA	Unicompartmental knee
2020	0.15	0.26	0.06	48.08	45.53	0.81	0.78	4.32
2021	0.32	0.22	0.07	53.13	36.91	2.16	1.09	6.11
2022	0.46	0.22	0.22	50.52	39.40	2.00	1.38	5.79
2023	0.42	0.19	0.13	47.90	42.99	2.21	1.31	4.86
2024	0.42	0.21	0.07	45.85	45.17	1.81	1.15	5.32

**Fig. 1:**
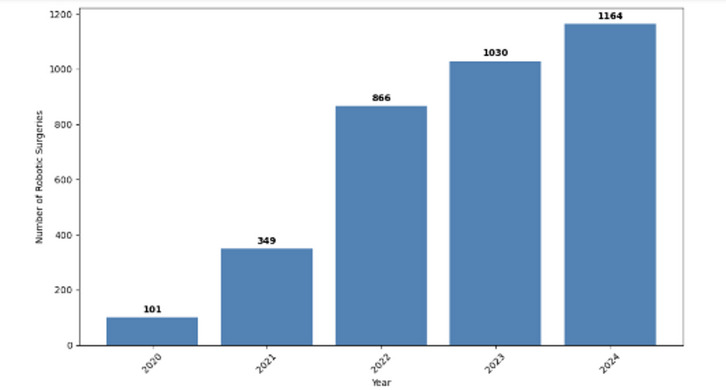
Number of robotic-assisted surgery per year

**Fig. 2:**
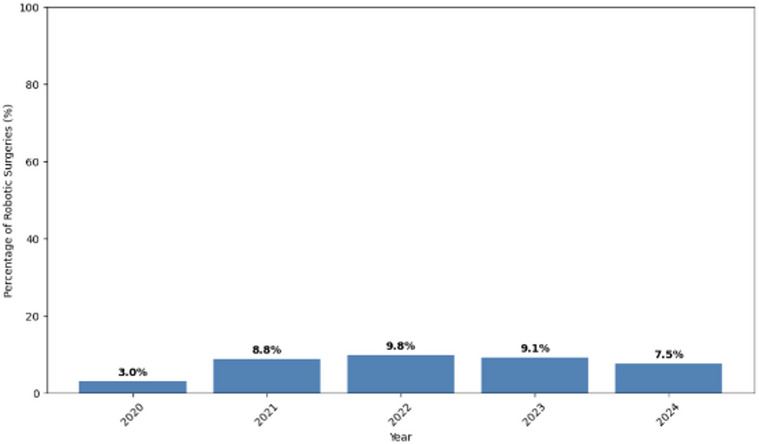
Percentage of all primary hip and knee replacements that used robotic-assisted surgery per year

The number of robotic-assisted joint replacement surgeries in NHS Scotland increased substantially from 101 procedures in 2020 to 1164 in 2024 (Fig. 1). This represents a tenfold increase over five years. However, despite this growth in absolute numbers, the percentage of robotic-assisted procedures peaked at 9.8% in 2022, with a slight decline to 7.5% in 2024 (Fig. 2).

**Fig. 3:**
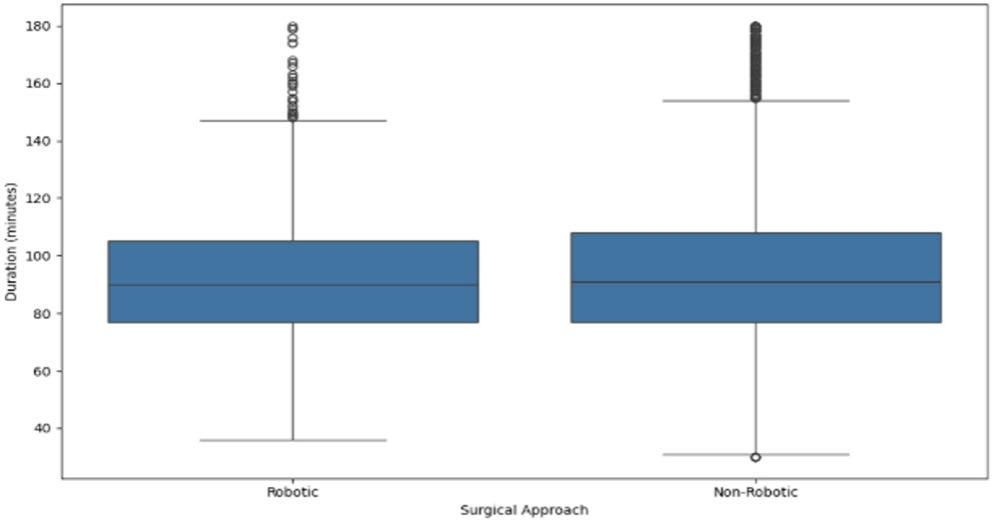
Surgery duration by robotic-assisted approach

In Fig. 3, no significant difference was observed in the median or mean operative duration between robotic-assisted and manual joint replacement surgeries. The median and mean surgery times were comparable across both groups. However, manual surgeries exhibited a greater number of short-duration outliers.

**Fig. 4:**
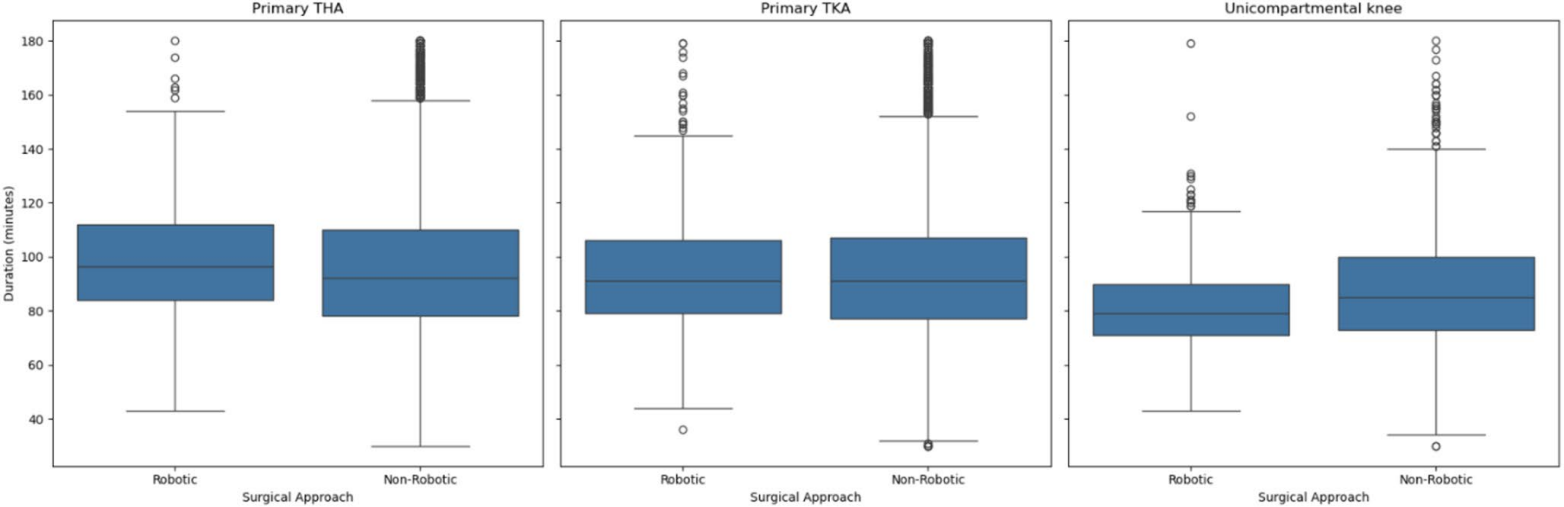
Surgery duration by joint type and robotic-assisted approach

Figure 4 illustrates that robotic-assisted surgeries demonstrated similar median operative durations compared to manual procedures for both hip and knee replacements. However, a narrower interquartile range (IQR) was observed in the robotic groups, which suggests a greater consistency in surgical time. For primary TKA, the robotic group had a median of 91 min (IQR: 27 min) compared with 91 min (IQR: 30 min) in the non-robotic group, and in unicompartmental knee replacements, 79 min (IQR: 19 min) versus 85 min (IQR: 27 min), respectively. Robotic cases also showed fewer extreme outliers, particularly for knee surgeries.

**Fig. 5:**
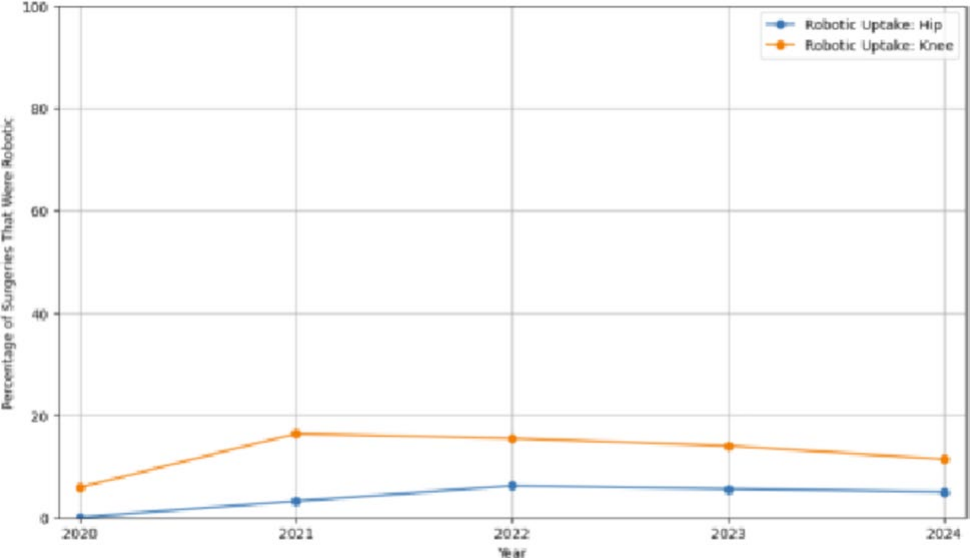
Robotic surgery uptake over time by joint type

The uptake of robotic-assisted hip and knee replacement increased from 2020 to 2022, with knee replacements consistently showing higher adoption rates than hip replacements throughout the period. Robotic knee procedures peaked around 2021–2022 at over 15% of all knee arthroplasties, while hip procedures remained under 10% in all years. After 2022, the adoption of robotic-assisted hip and knee replacement appeared to stabilise for both joints (Fig. 5).

**Fig. 6:**
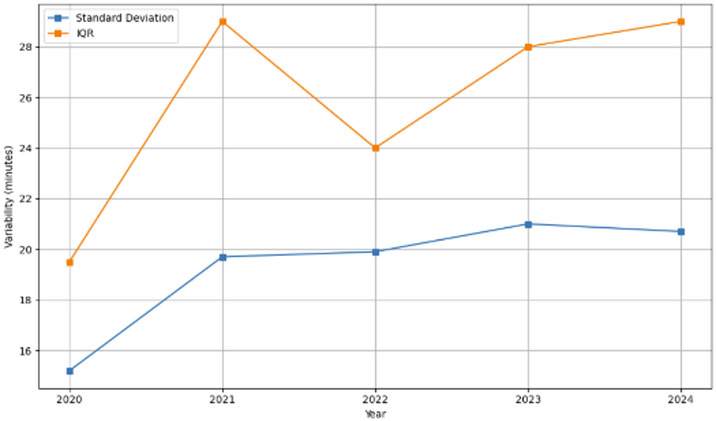
Variability in robotic surgery duration over time

Figure 6 shows the variability in robotic-assisted hip and knee replacement duration between 2020 and 2024. Both the standard deviation and interquartile ranges were lowest in 2021, which shows the most consistent surgical times during that year. Variability increased sharply in 2022 and remained relatively high through 2023 and 2024, which indicates greater inconsistency in surgery durations in later years.

Out of 23 hospitals included in the dataset, only six recorded robotic-assisted joint replacement surgeries between 2020 and 2024. As shown in Table 2, Hospital 2 and Hospital 5 (hospital names anonymised for reporting) performed the vast majority of cases, together accounting for over 97% of all robotic procedures. The remaining hospitals, not listed in Table 2, reported no robotic-assisted hip and knee replacement during the study period. This highlights a clear disparity in access to robotic surgical technology across NHS Scotland, likely driven by differences in funding availability, infrastructure capacity, and institutional readiness to implement and support such systems.

**Table 2:** Total Number of Robotic-Assisted joint replacement surgeries by hospital (2020–2024)

Hospital Code	Hospital 1	Hospital 2	Hospital 3	Hospital 4	Hospital 5	Hospital 6
Number of robotic surgeries	18	1088	11	8	2571	1

To control for contextual factors at the hospital level, we examined the two hospitals that performed more than 1000 robotic-assisted surgeries over the 4-year study period (Hospital 5: 2571 cases; Hospital 2: 1088 cases). In Hospital 5, robotic cases had a median duration of 90 min (IQR: 26 min) compared with 88 min (IQR: 24 min) for non-robotic cases. In Hospital 2, robotic cases had a median duration of 89 min (IQR: 29 min) compared with 87 min (IQR: 27 min) for non-robotic cases. These results show only minimal differences in median surgical time between robotic and non-robotic procedures within each hospital.

## Discussion

Findings from this study indicate that robotic-assisted hip and knee replacements are rapidly increasing across NHS Scotland, rising from just over one hundred cases in 2020 to nearly twelve hundred in 2024. NHS Golden Jubilee in Clydebank was the first hospital in Scotland to implement robotic technology routinely for total and partial knee replacements, initially projecting that approximately 300 patients would benefit within the first year of implementation. This early institutional support provided a foundation for the broader adoption of robotic-assisted knee replacement procedures across the country [11].

The fastest growth was specifically observed in knee replacement procedures, with uptake surpassing hip replacement surgeries in every year. This may be due to greater technical suitability or earlier integration of robotics in knee arthroplasty workflows, as robotic systems were initially developed and approved for use in knee replacements [7]. Technically, knee arthroplasty benefits from robotic assistance because precise alignment and soft-tissue balancing are critical to outcomes, and semi-active robotic platforms help optimise both. In a prospective cohort, robotic-arm assisted TKA showed lower pain, reduced analgesia use, faster straight-leg raise, greater knee flexion at discharge, and a shorter median time to discharge (77 h vs. 105 h) compared with conventional jig-based TKA [12]. Studies have also shown that robotic-assisted TKA results in higher accuracy and fewer alignment outliers, which can improve early clinical outcomes [13]. Additionally, the reproducibility of outcomes and consistent performance across procedures have made robotic knee replacements more appealing to surgeons [14].

However, following this period of rapid expansion, the rate of adoption now appears to have stabilised within this dataset. This relative plateau in robotic-assisted hip and knee replacements has been observed in recent years. This trend may reflect limited system availability and perceptions of only modest benefit compared with conventional techniques. Similar stabilisation has been described in other surgical domains, where an initial surge in adoption is followed by a period of consolidation as practical and cost–benefit considerations become more apparent. A comparable trajectory was seen with laparoscopic surgery, where rapid early adoption eventually plateaued as technical limitations and practical challenges emerged [15].

In terms of efficiency, the previously discussed clinical and technical benefits—combined with the fact that robotic-assisted hip and knee replacements are often performed by experienced surgeons—appear to translate into greater procedural consistency at the service level. Within NHS Scotland, robotic-assisted hip and knee replacement showed broadly comparable median operative durations to conventional joint replacements but consistently demonstrated lower variability and fewer outliers. This partly aligns with previous studies reporting that operative times were similar between robotic-assisted and manual knee replacements, with only marginal differences that were not clinically significant [16, 17]. This absence of major time savings likely reflects the setup and calibration required for robotic systems during early implementation, as well as the learning curve faced by surgical teams. As previously observed, efficiency in robotic-assisted arthroplasty tends to improve after approximately 15 cases [18]. However, variation in operative performance may be influenced by case complexity, surgeon experience, and institutional familiarity with robotic workflows [12, 19].

While the increasing use of robotic-assisted hip and knee replacement is a positive development, its adoption remains limited across the system. Hospital-level data confirm that the uptake of robotic-assisted hip and knee replacement in NHS Scotland is highly concentrated in a small number of centres. This uneven distribution is likely influenced by the substantial cost of acquiring and maintaining robotic platforms such as MAKO (Stryker) and ROSA (Zimmer Biomet). The MAKO SmartRobotics system costs between £500,000 and £1.5 million, including the robotic arm, planning software, and installation. Additional ongoing costs include annual maintenance, consumables, and specialised staff training [20]. Similarly, the ROSA Knee System carries comparable financial demands, including the robotic unit, operating software, and recurring operational expenses (Zimmer Biomet). These high costs limit adoption to larger and well-funded hospitals, often excluding smaller or resource-constrained sites [20]. As a result, access to robotic-assisted hip and knee replacement remains concentrated in a small number of centres. In addition to financial barriers, workforce capability and strategic allocation also play a role in restricting access. Robotic-assisted hip and knee replacement requires highly trained surgeons and specialist support teams, meaning that only centres with appropriate staffing and experience can maintain safe and efficient use of the technology [21]. These barriers may contribute to disparities in availability across the NHS and limit the broader implementation of this technology despite its clinical promise.

However, these challenges are unlikely to persist indefinitely. From a financial standpoint, while the initial costs of robotic systems remain high, experience from other technological domains suggests that prices typically decrease over time as markets mature. As more competitors enter the field and platforms mature, robot prices are expected to fall—by roughly 20–30% over the next five years—with some vendors moving to subscription pricing models [22]. Furthermore, many NHS trusts currently could lease or rent robotic systems rather than purchase them outright, which can mitigate upfront capital costs and accommodates rapid technology updates [23]. Also, Recent evidence indicates that robotic-assisted total hip arthroplasty can generate lower 90-day episode-of-care costs than manual techniques, driven by shorter length of stay and reduced post-index hospital use [24].

Regarding workforce barriers, while robotic-assisted hip and knee replacement is currently concentrated among experienced surgeons, its most transformative potential may lie in standardising outcomes across varying levels of surgical expertise. Robotic platforms enable precise and consistent implant positioning, thereby reducing variability between high- and low-volume surgeons [7]. Moreover, the learning curve for robotic systems is relatively short—estimated at 12–15 cases—and primarily affects time efficiency rather than complication rates [25]. By enhancing consistency and reducing operative variability, robotic systems can improve training efficiency, minimise outlier cases, and help achieve equitable surgical outcomes across the NHS.

In NHS Scotland, the strategic placement of robotic systems in high-volume or tertiary centres may maximise utilisation and return on investment, ensuring effective use of limited resources. However, this centralised approach risks restricting access for patients in regions without established robotic programmes. In the present analysis, robotic procedures represented fewer than 10% of all joint replacements, indicating a relatively low adoption rate. Careful and equitable scaling will therefore be essential to ensure that the benefits of robotic technology reach patients across all regions without creating disparities in access or care quality. Achieving this will require long-term planning, sustainable funding models, and coordinated national policies to promote equitable access to advanced surgical technologies.

Moreover, improving the distribution of robotic systems is not only a matter of equity but also of efficiency. The increasing procedural consistency observed in robotic-assisted hip and knee replacement suggests potential service-level advantages. Broader adoption could enhance workflow predictability and streamline theatre scheduling even if average operative durations remain similar to traditional techniques [26]. To realise these benefits, investment in staff training, equitable distribution of robotic platforms, and continuous outcome monitoring will be critical [21]. Ultimately, improved procedural predictability and reduced variability support wider NHS goals of operational efficiency, optimised patient flow, and consistent, high-quality surgical care [16].

## Limitations

One of the limitations in this study is the current classification of robotic and computer-assisted surgeries within Scottish national datasets. The existing coding system does not clearly distinguish between manual, navigated, and different types of robotic procedures. As a result, navigated procedures, which are computer-assisted, are grouped with manual surgeries. This lack of distinction may confound comparisons between robotic and manual cohorts, as navigated cases also incorporate elements of technological assistance that differ from conventional manual techniques.

This analysis is also limited by the absence of data on patient complexity, comorbidities, and postoperative outcomes, as well as the lack of a comprehensive cost-effectiveness assessment. As such, further work is needed to evaluate the broader clinical and financial impact of robotic-assisted hip and knee replacement in Scotland.

Another limitation of this analysis is the possible influence of implant design and manufacturer-specific factors on outcomes. Robotic and navigated systems are often tied to implant suppliers (for example, MAKO with Stryker and ROSA with Zimmer), making it difficult to separate the effect of the technology from that of the implant itself. In addition, the use of closed-platform robotic systems that are linked to specific implant manufacturers may limit wider uptake of robotic-assisted procedures and influence observed adoption patterns across centres. Variations in implant geometry, instrumentation, and compatibility with robotic platforms may therefore influence operative performance and outcomes. While such differences were not captured in the present dataset, they represent an important confounder that should be considered in future research comparing robotic and manual arthroplasty techniques.

In addition, the analysis does not account for differences between image-based and non-image-based robotic systems, which represent another unmeasured source of variation. For example, while MAKO (the predominant robotic platform in Scotland) is CT-based, the ROSA system is mostly non-image based and functions more similarly to navigation. These differences may have an influence on outcomes, but could not be explored due to limitations in the available data.

Finally, the dataset did not include information on surgeon experience or trainee involvement during surgery. The presence of trainees or less experienced surgeons may influence operative duration and workflow efficiency, particularly in complex or technology-assisted procedures. The absence of surgeon- and trainee-level data therefore represents an additional unmeasured confounder in the assessment of operative efficiency.

## Future work

Future work should focus on improving data granularity and linkage to enable a more detailed evaluation of robotic-assisted hip and knee arthroplasty. In particular, the ability to reliably distinguish between different robotic platforms, including image-based and non–image-based systems (such as MAKO, ROSA, and other emerging technologies), would allow quantification of platform-specific utilisation and comparison of efficiency and outcomes across systems. In addition, linking robotic platform data with implant manufacturer information would help separate the effects of surgical technology from implant-related factors and enable manufacturer-specific analyses.

Another important area for future research is the investigation of learning curve effects, which would require access to surgeon- or unit-level case sequencing. This would allow assessment of whether operative time decreases after a defined number of robotic cases. In parallel, capturing information on trainee involvement would allow future studies to assess both the prevalence of training cases and the impact of trainees on operating room efficiency. Similarly, more detailed theatre-level data, including operating room staffing and workflow characteristics, would support evaluation of how team composition influences efficiency.

Finally, improved and more complete capture of postoperative outcomes would strengthen future analyses. This includes more comprehensive recording of 30-day readmission outcomes to allow robust comparison between robotic-assisted and non-robotic procedures, as well as reliable coding of complication-specific events such as hip dislocation following total hip replacement. Together, these enhancements would support a more comprehensive assessment of the safety, efficiency, and clinical impact of robotic-assisted arthroplasty in routine practice.

## Conclusion

This study found that while robotic-assisted joint replacement surgeries have increased across NHS Scotland between 2020 and 2024, their use remains concentrated in a small number of centres. Operative durations were similar to conventional procedures, but reduced variability suggests potential benefits for theatre efficiency. Despite these advantages, broader adoption will require targeted investment in training, infrastructure, and equitable access. This is already occurring in the UK, as NICE has reported that 11 new robotic systems are currently being introduced, including next-generation image-based, imageless, and semi-autonomous platforms [20]. These emerging systems may offer additional advantages such as reduced setup time, enhanced intraoperative feedback, and broader implant compatibility.

In the future, with appropriate governance, data integration, and long-term planning, robotic-assisted hip and knee replacement has the potential to play an important role in improving surgical precision, operational efficiency, and service delivery across the NHS.

## Data Availability

The data used in this study come from the anonymised ARISE dataset provided by NHS Scotland. These data are not publicly available due to governance restrictions.Access to the dataset was granted through approved data-sharing forms, and therefore the data cannot be shared publicly.
